# The Conserved *nhaAR* Operon Is Drastically Divergent between B2 and Non-B2 *Escherichia coli* and Is Involved in Extra-Intestinal Virulence

**DOI:** 10.1371/journal.pone.0108738

**Published:** 2014-09-30

**Authors:** Mathilde Lescat, Florence Reibel, Coralie Pintard, Sara Dion, Jérémy Glodt, Cecile Gateau, Adrien Launay, Alice Ledda, Stephane Cruvellier, Jérôme Tourret, Olivier Tenaillon

**Affiliations:** 1 Institut National de la Santé et de la Recherche Médicale (INSERM), Unité Mixte de Recherche (UMR) 1137, Paris, France; 2 Laboratoire de Microbiologie, Hôpital Jean Verdier, Assistance Publique-Hôpitaux de Paris, Bondy, France et Université Paris Nord, Sorbonne Paris Cité, Paris, France; 3 UMR 1137, Université Paris Diderot, Sorbonne Paris Cité, Paris, France; 4 Laboratoire de Génomique Comparative, Centre national de la Recherche Scientifique (CNRS) UMR 8030, Institut de Génomique, Commissariat à l'énergie atomique et aux énergies alternatives (CEA), Genoscope, Evry, France; 5 Département d'Urologie, Néphrologie et Transplantation, Hôpital Pitié-Salpêtrière, Assistance Publique-Hôpitaux de Paris et Université Pierre et Marie Curie, Paris, France; Institut Pasteur, France

## Abstract

The *Escherichia coli* species is divided in phylogenetic groups that differ in their virulence and commensal distribution. Strains belonging to the B2 group are involved in extra-intestinal pathologies but also appear to be more prevalent as commensals among human occidental populations. To investigate the genetic specificities of B2 sub-group, we used 128 sequenced genomes and identified genes of the core genome that showed marked difference between B2 and non-B2 genomes. We focused on the gene and its surrounding region with the strongest divergence between B2 and non-B2, the antiporter gene *nhaA*. This gene is part of the *nhaAR* operon, which is in the core genome but flanked by mobile regions, and is involved in growth at high pH and high sodium concentrations. Consistently, we found that a panel of non-B2 strains grew faster than B2 at high pH and high sodium concentrations. However, we could not identify differences in expression of the *nhaAR* operon using fluorescence reporter plasmids. Furthermore, the operon deletion had no differential impact between B2 and non-B2 strains, and did not result in a fitness modification in a murine model of gut colonization. Nevertheless, sequence analysis and experiments in a murine model of septicemia revealed that recombination in *nhaA* among B2 strains was observed in strains with low virulence. Finally, *nhaA* and *nhaAR* operon deletions drastically decreased virulence in one B2 strain. This effect of *nhaAR* deletion appeared to be stronger than deletion of all pathogenicity islands. Thus, a population genetic approach allowed us to identify an operon in the core genome without strong effect in commensalism but with an important role in extra-intestinal virulence, a landmark of the B2 strains.

## Background

Comparative genomics has unraveled the dynamics of microbial genome evolution [1]. The extent of lateral gene transfer has appeared to be one of the most striking characteristics of this dynamics. These transfers impact the most studied phenotypes of bacteria: antibiotic resistance and virulence. For example, horizontally acquired clusters of genes found in pathogenicity islands (PAI) have been shown to be involved in virulence [2]. Yet adaptation may also occur through mutations in genes present in the whole species, or core genes. This topic has been far less studied, despite the large potential for adaptation through mutations in core genes that experimental evolution has revealed [3]. The principal reason is that, because of the limited amount of recombination, most mutations are linked and therefore identifying the ones that are involved in adaptation is challenging. Nevertheless, if selective pressure is strong enough as in the case of antibiotic resistance or some cases of virulence, a few mutations in core genes have been found to be involved in adaptation [4], [5]. In the present paper, we want to extend such an approach and try to identify some core genes that may contribute to the functional divergence between phylogroups in the *Escherichia coli* species.

*E coli* is a versatile bacterium, both retrieved in the environment and known as a widespread gut commensal of vertebrates, especially humans. *E. coli* is also a pathogen which is responsible for more than 1 million deaths a year due to intra and extra-intestinal diseases. In the wild, its population size has been estimated to more than 10^20^ bacteria [6]. The species has a clonal structure, and is subdivided in seven phylogenetic groups A, B1, B2, C, D, E and F [7]. These groups are not randomly distributed. Indeed, previous studies have shown a correlation between phylogeny and virulence in *E*. *coli*, with most extra-intestinal pathogenic *E. coli* (including urinary tract infection and meningitis associated strains) belonging to phylogenetic group B2 [8], [9]. Moreover the prevalence of the different groups among commensal strains varies largely across host species and even across populations of a given host species. For instance, B2 strains not only are commonly isolated from extra-intestinal infections, but also appear to be efficient commensals frequently retrieved in wild animals and humans [10]. In humans, the prevalence of B2 commensals varies drastically according to populations, being low in tropical countries and high in developed countries [6]. It appears that the frequency of B2 carriage has increased over the last 30 years (e.g. from 20 to 40% in France) a worrying observation knowing their extra-intestinal pathogenic potential as well as their implication in colon cancer [11], [12]. Unraveling the bases of this success in the commensal habitat is of medical relevance. Moreover, the inactivation of some of the virulence factors have been shown to reduce the ability to colonize the gut [13], comforting the idea that extra-intestinal virulence is a by product of commensalism [14].

Whether an *E. coli* strain behaves as a commensal or a pathogen is determined by an extremely complex balance between many factors: immune status of the host, production of virulence factors by the bacterium, portal of entry, inoculum, and the genetic background of the bacterium to cite some important ones. The latter appears to be essential for acquisition and expression of virulence factors [15]. Yet, the alleles involved in the specificities of the different group of strains remains largely unknown, especially in the primary habitat, the gut of vertebrates where *E. coli* is mainly a commensal strain.

**Figure 1:**
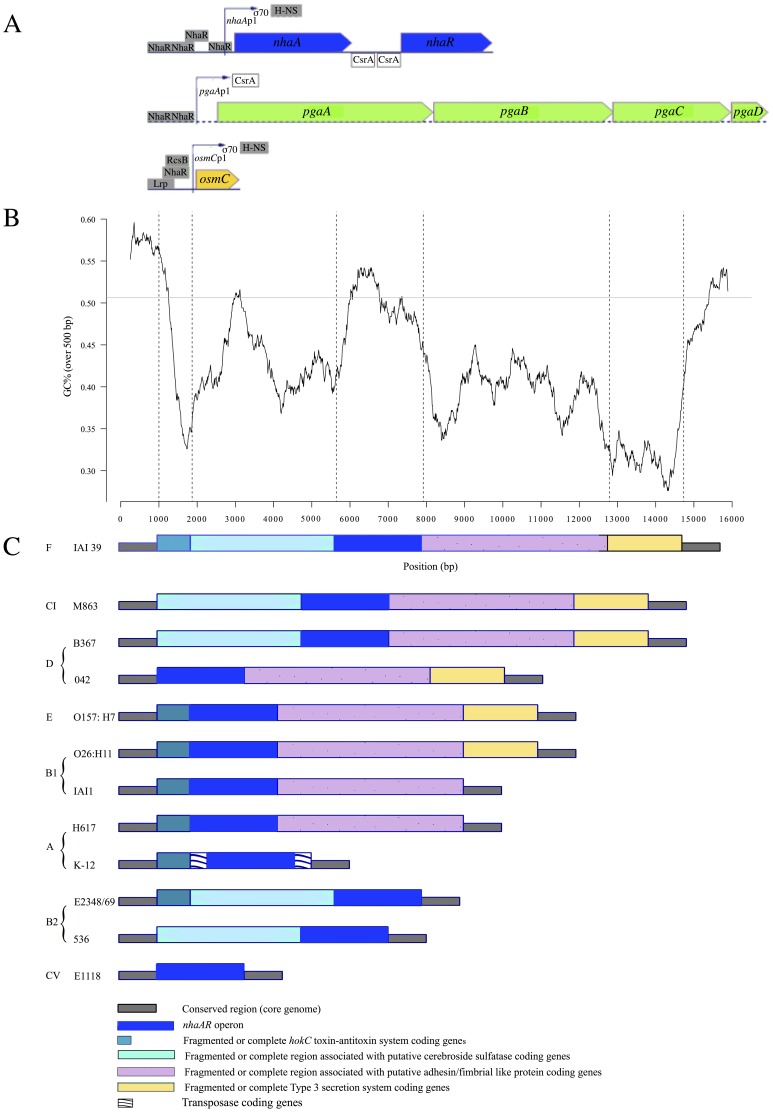
Genomic organization of *nhaAR* region. (A) Genomic representation of *nhaAR* and other operons under NhaR regulation in K-12 *E.coli* strain from http://www.ecocyc.org. All transcription or translation regulators are indicated. (B) GC percent along IAI39 *nhaAR* region (black line) and mean core genome GC percent (gray line) (C) Organization in various modules of *nhaAR* region. The modular organization of the region was defined using synteny breaks between 10 pathogenic and commensal *E. coli* and 2 clades strains from various phylogenetic groups including K-12 and H617 (two group A commensal strains), IAI1 and O26:H11 (two group B1 commensal strains), B367 (a group D commensal strain), 042 (an enteroaggregative group D, E2348/69 (an enteropathogenic group B2 strain), 536 (an extra-intestinal pathogen group B2 strain), O157:H7 Sakaï (an enterohemorrhagic group E strain). The two *Escherichia* clades (C) used were M863 (CI) and E1118 (CV). Five homologous modules have been defined, *nhaAR* operon being the third. Dark green: Fragmented or complete *hokC* toxin-antitoxin system coding genes; turquoise: Fragmented or conserved region associated with putative cerebroside sulfatase coding genes; blue: *nhaAR* region; pink: Fragmented or conserved region associated with putative adhesin/fimbrial like protein coding genes; yellow: Fragmented or conserved Type 3 secretion system coding genes. IS are also indicated.

To perform comparative genomics on a large scale, *E. coli* is an organism of choice with 128 complete genomes available. Based on that collection, we identified several candidate genes showing the highest divergence between B2 strains and the rest of the species. Our aim was not just to provide a list of genes but also to perform functional tests. Therefore, we focused our attention on the region centered on the gene *nhaA* as this was the candidate with the highest divergence opening the path to functional assays. *nhaA* is part of an operon coding for a sodium proton antiporter which is known to be responsible for pH and sodium homeostasis in *E. coli* [16] (Figure 1A). The aims of this study were (i) to identify markers of differentiation of the B2 phylogenetic group (ii) to perform population genetic analysis on the sequences of the candidate (iii) to identify a potential biological role for this marker *in vitro*, and (iv) to test its potential role *in vivo* in a mouse colonization assay and a mouse septicemia model.

## Materials and Methods

### Ethics statements

All *in vivo* experiments were realized in accordance with the ARRIVE guidelines. The murine septicemia was conducted following European and National regulations for housing and care of laboratory animals after pertinent review and approval by the Bioethics Committee at Santiago de Compostela University and by the French Veterinary Services (certificate number A 75-18-05). The murine gut colonization model was conducted after approval by the Debre-Bichat Ethics Committee for Animal Experimentation (Protocol Number 2012-17/722-0076) in accordance to the European Decret and French law on the protection of animals. All possible measures were taken to minimize animal suffering and to ensure animal welfare. When necessary, animals were sacrificed by lethal intra-peritoneal injection of phenobarbital after volatile anesthesia with sevoflurane.

### Bacterial strains

All *E. coli* strains and plasmids are listed in Table S1. Strains have been chosen for their representativity of the phylogeny and their wide array of phenotypes as they were isolated from commensal, extra-intestinal and intra-intestinal pathogenic situations.

### Inactivation of the *nhaAR* region and control experiment

Inactivation of *nhaAR* was performed using the modified method described by Datsenko *et al.* [17]. We first obtained a PCR product using the K-12, TA249 and IAI1 strains, with primers WanF_nonB2_nhaAR for all strains and WanR_K12TA249_nhaAR for K-12 and TA249, and WanR_IAI1_nhaAR for IAI1. The same PCRs were done using CFT073, 536 and TA014 strains with primers WanF_B2_nhaAR and WanR_B2_nhaAR. We also performed the inactivation of *nhaA* and *nhaR* genes in 536 strain using the primers WanF_B2_nhaA with WanR_B2_nhaA and WanF_B2_nhaR with WanR_B2_nhaAR for *nhaA* and *nhaR* disruption, respectively. PCR products contained (i) the FLP recognition target FRT-flanked chloramphenicol resistance gene (*cat*) and (ii) the 50-bp sequences homologous to the 5′ and 3′ flanking regions of *nhaAR, nhaA* and *nhaR* for each corresponding strain. Inactivation of the *nhaAR* operon, *nhaA* and *nhaR* genes were confirmed by PCR using the following primers: verifWanF_nonB2_nhaAR and verifR_nonB2_nhaAR for non-B2 strains and the primers verifWanF_B2_nhaAR and verifR_B2_nhaAR for B2 strains, targeting sequences upstream and downstream from the *nhaAR* operon; and c1 and c2, targeting sequences within *cat* gene; All strains obtained and primers used are listed in Tables S1 and S2.

### Complementation of the *nhaAR* region and control experiment

Complementation of the strain 536Δ*nhaAR* by the *nhaAR* region was performed using the GC Cloning & Amplification Kit (pSMART GC LK vector) (Lucigen, Middleton, WI). Briefly the *nhaAR* region including the promoter region were amplified from the 536 strain using the primers cp_536F and cp_536R targeting sequences 200 bp upstream *nhaA* start and 200 bp downstream *nhaR* stop, respectively. The *nhaA* gene was amplified using cp_536F and cp_536DnhaA_R targeting sequence 200 bp downstream *nhaA* stop. The fragments were then separately cloned into the blunt cloning site in the pSMART GC LK vector. The plasmids bearing the *nhaAR* region or *nhaA* gene were then electroporated in 536Δ*nhaAR* strain and or 536Δ*nhaA*. The complementation experiments were confirmed by PCR using the primers cp_536F and cp_536R. We also incorporated the pSMART GC LK vector in 536Δ*nhaAR* and 536Δ*nhaA* strains as controls. All strains obtained and primers used are listed in Tables S1 and S2.

### Genomic environment analysis

The MicroScope platform [18] was used for comparative analysis of genetic sequences surrounding *nhaAR*. The MicroScope platform allows comparative analysis of available *E. coli* and closely related genomes, with visualization of *E. coli* genome annotations enhanced by a synchronized display of synteny groups in the other genomes chosen for comparison.

### Reconstruction of the phylogenetic tree

The 121 *E. coli* genomes from the MicroScope website were included [23]. A maximum-likelihood phylogenetic tree was reconstructed with the PHYML software [19] using the concatenated multi locus sequence typing (MLST) sequences on the one hand, and *nhaA* sequences on the other hand. We used the MLST Pasteur scheme [7].

### Sequence alignments and study of recombination

We compared 128 *E. coli*/*Escherichia clade* [20] *nhaAR* sequences of 2303 bp by sequence alignment using ClustalW software [21]. Observation of traces of recombination was performed on the 1167 bp of *nhaA* sequences by comparison between the sequences of B2 strains showing long branches in the phylogenetic tree sequences, other B2 strain consensus sequence, non-B2 strain consensus sequence and *Escherichia* clade sequences. Amino-acid sequences inferred from the nucleotide sequences of the *nhaAR* region were also analyzed. After the generation of the maximum likelihood tree (see above), amino-acid substitutions for each branch of the *nhaAR* tree were identified by comparison of consensus sequences between different branches using the BIOEDIT software [22].

### Analysis of genomic environment of *nhaAR*

The genomic environment was observed using the synteny breaks between two clades and 10 *E. coli*. Of these, 5 were pathogenic strains, including E2348/69 (an enteropathogenic group B2 strain), 536 (an extra-intestinal pathogen group B2 strain), 0157:H7 Sakaï (an entero-hemorrhagic group E strain), 042 (an entero-aggregative group D strain) and IAI39 (an extra-intestinal group F strain), whereas 5 were commensal strains including K-12 and H617 (two group A strains), IAI1 and O26:H11 (two group B1 strains) and B367 (a group D strain). The two clades were M863 (*Escherichia* clade I) and E1118 (*Escherichia* clade V). It appeared that this region had a composite structure,*i.e.* it is made up of five modules that are present or absent in the different strains. We then classified the strains according to the maximum number of regions present to retrace a parsimonious history of loss and gains of these modules.

### Flow cytometry

The wild type and their Δ*nhaAR* isogenic mutants strains K-12, IAI1, TA249, CFT073, 536 and TA014 in which the plasmids from the Zaslaver collection have been introduced (Table S1) were compared using the wild type strains K-12, IAI1, TA249, CFT073, 536 and TA014 as controls. The Zaslaver collection is a bank of *E. coli* K-12 strains in which reporter plasmids bearing the Gfp protein under control of the promoter regions of each gene available was introduced [23] Plasmids were extracted using the plasmid min kit extraction kit (Sigma). The experiments were conducted as described elsewhere [24].

### Growth curves

For the comparative growth assays, K-12, IAI1, TA249, CFT073, 536 and TA014 wild type strains and their mutants, K-12Δ*nhaAR*:Cm, IAI1Δ*nhaAR*:Cm, TA249Δ*nhaAR*:Cm, CFT073Δ*nhaAR*:Cm, 536Δ*nhaAR*:Cm and TA014Δ*nhaAR*:Cm were grown at 37°C in 2 media: Luria Bertani (LB) and Davies minimum medium (DM) with glucose (NaH_2_PO_4_ 33.9 mmol/L, Na_2_HPO_4_ 31.1 mmol/L, (NH4)_2_SO_4_ 20 mmol/L, MgSO4 7 H2O 0.3 mmol/L, KCl 40,2 mmol/L, FeCl3 70 µmol/L, glucose 20 mmol/L), each media was adjusted at pH 7 and pH 8.5 with MOPS and TAPS, respectively (Sigma), DM was also adjusted at pH 8 and at NaCl: 170 mmol/L and 300 mmol/L (Table S3). LB is a complex medium, whereas DM is a minimal medium with only one source of carbon. All the studied strains were grown overnight (O/N) in LB medium in deep-weel plates at 37°C with constant shaking at 280 rpm. O/N cultures were pre-diluted at 1/100 in saline buffer and strains were inoculated in four different wells each at 1/100 in a Costar 96 flat-bottomed well plate. Growth was recorded by an Infinite 200 Tecan, which measured the OD600 in each well every 5 minutes at 37°C, while shaking for 24 hours. Growth assays were repeated 3 times. The maximum growth rate (MGR in s^−1^) was computed from growth curves obtained by Tecan. Briefly, OD600 were collected, log-transformed, and smoothed with a spline function. The MGR was defined as the maximum value of the derivative of the smoothed growth curve. The doubling times (DT) (in mn) have then been computed as followed. DT =  Log2/(MGR*60). All DTs were compared by strain and by medium using the Welch test.

### Murine Septicemia model

A mouse model of systemic infection [8] was used to assess the intrinsic virulence of strains SE15, H001, TA103 and TA435 which showed traces of recombination. To compare intrinsic virulence of B2 strains with a recombinant *nhaAR* operon and B2 strains without trace of recombination at the operon locus we used previous results of intrinsic virulence of strains CFT073, 536, F11, S88, APEC01, UTI89, LF82 and B2S [14], [25]. In order to avoid a day-of-experiment bias, K-12 and 536 were included in all experiments as negative and positive controls of intrinsic virulence, respectively. To test the effect of the deletion of the *nhaAR* operon on intrinsic virulence of *E. coli* B2 strains in different genomic backgrounds, we tested CFT073, CFT073Δ*nhaAR*:Cm and CFT073Δ*nhaAR* strains, a mixture of equal quantities of CFT073 and CFT073Δ*nhaA*:Cm and a mixture of equal quantities of CFT073Δ*nhaAR* and CFT073Δ*nhaAR*:Cm to test for the cost of the antibiotic resistance. We also tested 536 and 536Δ*nhaAR* strains, a mixture of equal quantities of 536 and 536Δ*nhaAR*:Cm. To decipher which gene was responsible for virulence attenuation in the operon, we tested the deleted mutant strains 536Δ*nhaA and* 536Δ*nhaR*. Finally, we tested the complemented strains 536Δ*nhaAR* pGC*nhaAR*, 536Δ*nhaAR* pGC*nhaA*, 536Δ*nhaA* pGC*nhaAR* 536Δ*nhaA* pGC*nhaA*. The complemented strains 536Δ*nhaAR* pGC and 536Δ*nhaA* pGC in which the deleted mutant strains have been complemented with an empty vector were used as control of empty vector cost in the murine model of septicemia. The experiments were conducted as described elsewhere [8]. Briefly, the ability of bacterial strains to cause sepsis was determined using 5-wk old female OF1 mice (Charles River, L'Arbresle, France). 10 mice per strain or mixture of strains tested were used. A total of 200 µl of a suspension of 109 bacteria/ml in saline buffer was inoculated by subcutaneous injection in the neck, and mortality was recorded during the following 7 days. For competition assays, spleens were aseptically collected after death, homogenized in 1 ml of saline buffer, and plated in serial dilutions on LB agar with or without appropriate antibiotic. For assays where strains were tested alone, spleens were aseptically collected after death, homogenized in 1 ml of saline buffer, and plated in serial dilutions on LB agar with or without appropriate antibiotic, colony were verified by PCR using cp_536F and cp_536R primers (Table S2).

### Mouse model of intestinal colonization

Intestinal colonization was assessed using a mouse model as described elsewhere [13]. Briefly, 6-wk old CD1 female mice (Charles River, L'Arbresle, France) treated with streptomycin were used. Five days before inoculation, was added to the sterile drinking water at a final concentration of 5 g/liter. Streptomycin was maintained throughout the whole experiment. Coliform-free mice were inoculated through oral gavage with 10^6^ bacteria in 200 µl of saline buffer. Every day post-inoculation, dilutions of weighed fresh feces resuspended in 1 ml of saline buffer were plated on LB agar with or without appropriate antibiotic. We studied 536 wild type strain and isogenic mutants 536Δ*nhaAR*:Cm, and 536Δ*nhaAR*. For each strain two mice were used. We also performed competition assays using a mixture of equal quantities of 536 wild type and 536Δ*nhaAR*:Cm to test the effect of deletion of the region on the gut colonization ability and also a competition between 536Δ*nhaAR*:Cm and 536Δ*nhaAR* to test the cost of *cat* resistance gene. For each competition four mice were used once.

### Statistical analysis

Population genetics analyses were performed using libsequence [26]. For phenotypic analysis, the values are given as medians (interquartile range) and, comparisons between strains were performed using either the Wilcoxon signed-rank test or the Kruskal-Wallis equality-of-populations rank test, unless specified otherwise. All statistics were computed using STATA (v10.0, College Station, TX, USA) or R (R Development Core Team, 2009, Vienna, Austria) and statistical significance was determined at a p-value of less than 0.05.

### RNA isolation

Total RNA extraction was performed on 536, 536Δ*nhaAR*, 536Δ*nhaR and* 536Δ*nhaA* after O/N culture during 18 h at 37°C in LB medium. Each culture for each bacteria was repeated three time. Total RNA was extracted using the hot phenol method. Residual chromosomal DNA was removed by treating samples with a Ambion TURBO DNA-free Kit DNase-treated RNA samples were quantified using a NanoDrop 1000 spectrophotometer (Thermo Scientific).

### Quantitative RT-PCR (qRT-PCR)

qRT-PCR experiments were performed using a KAPA SYBR One-Step qRT-PCR Kit (Kapa Biosystems) and a Lightcycler 480 (Roche) instrument with the program recommended by Kapa Biosystems. We applied the comparative CT quantification (ΔΔCt method) of qRT-PCR for comparing changes in gene expression of *nhaR* in the 536 deleted mutant strains. Relative quantification was performed using 16S*rRNA* as endogenous control gene. Each experiment was performed in duplicate.

## Results, Discussion and Conclusion

### Results

#### Genomic analyses

To identify genetic markers in the core genome that would differentiate the B2 phylogenetic group from the other groups, we scanned all genes in the core genome of 128 *E. coli*/*Escherichia clade* genomes. For each gene, we computed the number of fixed mutations between B2 and non-B2 and compared it with a Fisher test to the pooled core genome number. We studied the proportion of fixed sites compared to the total gene length or to the total number of polymorphism found in that gene. In both cases, the gene with the lowest p-value was *nhaA*, (p< 1e-62), the next gene being *ygbE* (p< 1e-33) a conserved gene of unknown function (Table S5). In this paper, we focused on the *nhaAR* operon, as *nhaA* is the first gene of the list, but also because it can be functionally assessed as it is a sodium proton antiporter involved in pH and sodium homeostasis [16].

**Figure 2:**
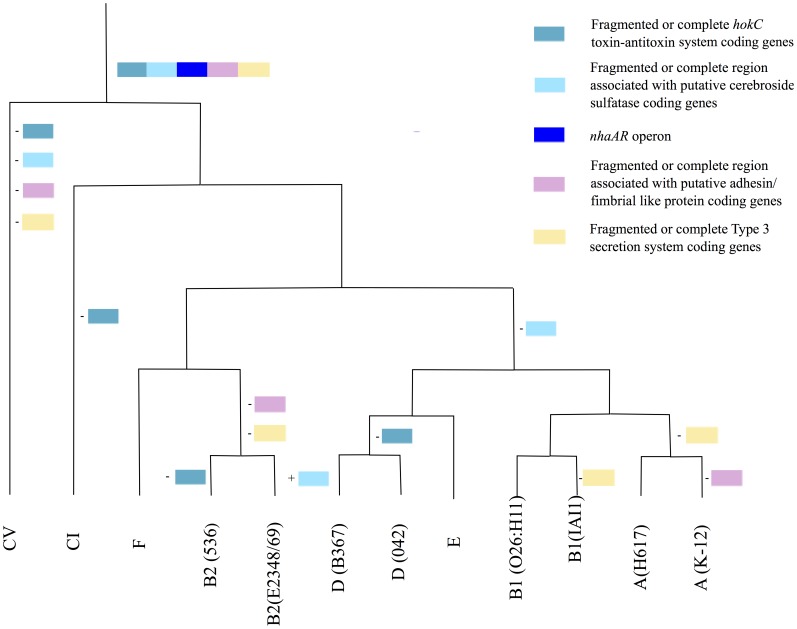
Multiple gains and losses of modules around *nhaAR* operon. A parsimonious scenario of gains and losses of the 5 modules defined in figure 1 is presented along the phylogenetic tree of the strains. – indicates losses and + acquisitions (colors of modules as in figure 1).

We first compared the genomic environment of the *nhaAR* operon in 10 *E. coli* strains and two *Escherichia* clades (clade I and clade V) (Figure 1BC). We defined 5 homologous fragments composing this region excluding fragments of transposases. Apart from the fragment including exclusively *nhaAR* operon, none of the other fragments were found in all strains, yet they were all present in strain IAI39. The GC content in the region was on average 42.67%, and differed significantly from the average genome GC content of 50.63% (p<0.05) (Figure 1B). This suggests that the region might have been acquired through horizontal gene transfer. Nevertheless, *nhaAR* had a GC content compatible with the genomic one. The pattern of gain/loss of the fragments surrounding *nhaAR* appeared to be quite dynamic (Figure 2). All the fragments seemed to have been lost and or gained multiple times along the phylogeny. *nhaAR* operon has therefore been maintained in the core genome despite highly dynamic surrounding regions, an observation that suggests an important contribution of this operon to *E. coli* niche adaptation.

**Figure 3:**
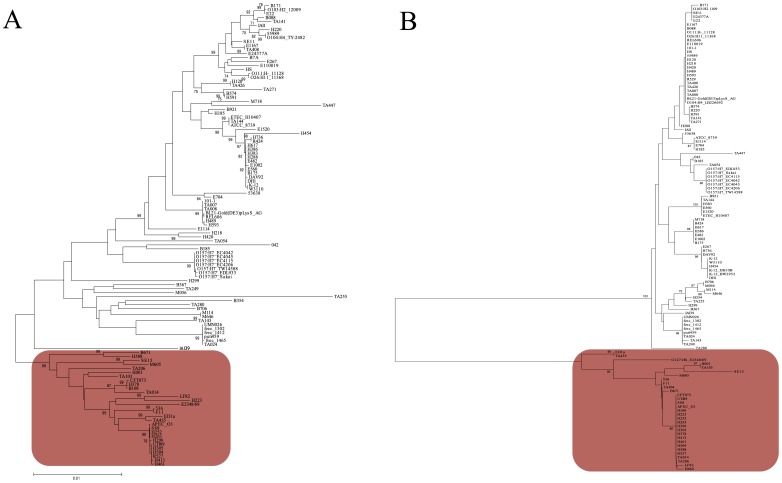
MLST and *nhaA* phylogenetic trees for 121 strains of *E. coli*. The trees were reconstructed from (A) multi-locus sequence typing of 8 partial housekeeping genes from the Pasteur scheme [7] representing the species phylogeny and (B) from the *nhaA* sequences using PHYML [19]. Bootstraps values are indicated. Strains studied and belonging to phylogenetic group B2 (red boxed) are indicated. Branches separating the B2 phylogenetic group strains from the other group strains are indicated in blue.

We reconstructed the phylogenetic tree of the *nhaA* gene from the 121 genomes of *E. coli* available in data banks (Table S4). Consistent with the screen used to identify *nhaA* region, we found that in the phylogenetic tree based on *nhaA* the branch leading to the B2 group of strains was much longer than what was found using the MLST genes of the Pasteur scheme [7] (Figure 3). There were 56 mutations that were fixed between the B2 and the non-B2 strains on a 1167 bp gene, or 4.8% of sites which contrast quite drastically with the whole genome average of 0.14%. This could be due to an accelerated evolution at this locus or to horizontal gene transfer or both. Yet, when we measured Ka/Ks (corresponding to the ratio of non synonymous mutation rate on the synonymous mutation rate) between B2 and non-B2 strains, a value between 0.01 and 0.02 was found. This means that synonymous mutations were in large excess compared to non-synonymous mutations. As we can exclude that selection of a succession of non-synonymous mutations was responsible for the long-branch, we favor horizontal gene transfer as the most likely explanation. The 5 to 10% divergence observed between B2 and non-B2 *nhaA* genes suggests that the transfer originated from a close species like an *Escherichia clade* and that this transfer may have been quite recent such that little recombination might have occurred subsequently between the B2 and the other strains. Accordingly, visual inspection of the *nhaA* sequences revealed that a few B2 strains (ED1a, SE15, E2348/69, H001, TA103, M605, and TA435) had three or more consecutive mutations that differed from the other B2, which can be considered as a trace of recombination. Similarly in the *nhaR* region, a long recombinant segment in strain M605 was responsible for most of the diversity within B2. When recombining strains were excluded from the analysis, *nhaR* appeared with an even stronger B2/non-B2 differentiation than *nhaA* with 13.0% of fixed differences compared to 5.8% for *nhaA*. Therefore the whole *nhaAR* operon and not just *nhaA* harbors a strong divergence between B2 and non-B2. Interestingly, while B2 strains are commonly isolated from extra-intestinal infections, none of the strains with sign of recombination have been isolated in extra-intestinal conditions. ED1a, SE15, H001, TA103, M605, TA103, TA435 were sampled in commensal conditions [14], [27], [28]. E2348/69 is an entero-pathogenic strain [29]. Even more strikingly, some of these strains are quite atypical B2 strains in terms of extra-intestinal virulence as they are non-killer in a mouse model of septicemia. For instance, ED1a strain belongs to the B2 subgroup VIII, a specific commensal subgroup never retrieved in extra-intestinal virulence conditions, specific to the human digestive track [30], and avirulent.

We compared consensus sequences of the 1167 bp *nhaA* gene and the 172 bp promoter region between B2 and non-B2 strains. We identified 98 mutations. Of these, 3 were non-synonymous, 2 deletions and 3 indels, and none of them were in a position described as important for the protein. The promoter region was highly conserved among the B2 with a single polymorphism out of 172 bp among the 28 B2 strains (Watterson estimate per base: 0.0015), and much more diverse in the non-B2 (28 polymorphic sites among 90 strains; Watterson estimate per base: 0.0321). Five of the mutations that differentiated the B2 and non-B2 were found in the NhaR1 and NhaR4 binding sites of NhaR regulator (Figure 1A). These mutations suggest variable level of expression between B2 and non-B2 strains.

We also looked at the *nhaR* coding region (905 bp) and the inter-genic region between *nhaA* and *nhaR* (60 bp), which is involved in post-transcriptional regulation of *nhaR* by CsrA [31] (Figure 1A). Thirteen non-synonymous mutations were found between B2 and non-B2 strains. Moreover, the hairpin-loop binding site used by CsrA to modulate NhaR regulation harbored 3 mutations. This lead us to hypothesize a differential expression of the *nhaAR* operon with potential consequences on the genes regulated by NhaR, *i.e. nhaA*, *pgaA* and *osmC*.

#### Phenotypic results

To assess whether *nhaAR* region is implicated in virulence or commensalism, we tested different phenotypes linked with pH and osmolarity that could differentiate B2 and non-B2 strains. We first wanted to investigate the expression level of the operon in the two backgrounds. We used 3 strains of each group and introduced reporter plasmids bearing the Gfp protein under control of the promoter regions of *nhaA* and *osmC*. Both promoters are under NhaR control and can be used to monitor repression it imposes by flow cytometry. However, Gfp expression under *nhaA* promoter was too low in all tested conditions, and fluorescence controlled by *osmC* promoter did not show any B2/non-B2 difference across all the conditions tested (data not shown).

**Figure 4:**
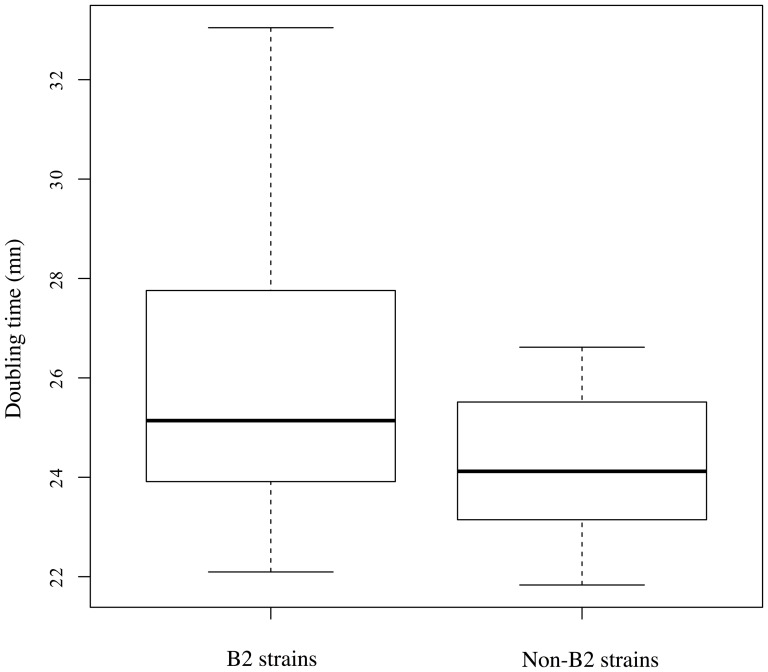
Non B2 grew faster than B2 in high pH high osmolarity. Boxplots of the doubling times (DT) in minutes of 12 B2 and 10 non-B2 representative strains of *E. coli* in LB, pH 8.5 with 350 mmol/L of sodium. We found a significant difference between B2 strains and non B2 strains using a Welch test (p = 0.001).

We then focused on growth curves in different media. Doubling times of 9 B2, 10 non-B2 and 3 B2 strains showing traces of recombination in *nhaAR* region were cultured in different media (LB and minimum growth medium with glucose as carbon source) at different pH (7, 8 and 8.5) and at different sodium concentrations (170 and 350 mmolL) (Figure 4). A statistical difference in growth between B2 and non-B2 strains was observed in LB at pH 8.5 and concentration of sodium of 350 mmol/L (p = 0.001). Interestingly, these conditions are the ones in which *nhaA* is induced [32]–[34]. The 4% difference observed in division time seems modest but corresponds to a drastic selective advantage: the ratio of non-B2 to B2 would double every 10 hours of competition in that media and result in a 150 fold increase of non-B2 over B2 in 3 days. However, this does not prove a direct contribution of *nhaAR* to this difference. We therefore looked for some direct implication of *nhaAR* operon by studying knock out mutants.

Effect on growth of the deletion of the operon *nhaAR* in 3 strains of B2 group (CFT073, 536 and TA014) and 3 strains from other groups (K-12, IAI1 and TA249) was studied in the same media. Most of mutants were not able to grow with minimum media at pH 8.5 as observed by others [33], we then used pH 8 to analyze growth in the minimum media. We analyzed several statistics of growth (MGR and maximal optical density) and compared the deletion mutants in absolute terms (MGR) or relative to their wild-type strain (change in MGR). The 3 B2 strains had a very comparable growth. In contrast, the non-B2 strains had very different growth characteristics, and one of the three strains had a pattern similar to the B2. As a result, there was no significant differential effect of the *nhaAR* deletion between B2 and non-B2 on growth in the tested conditions.

#### Mouse models

**Figure 5:**
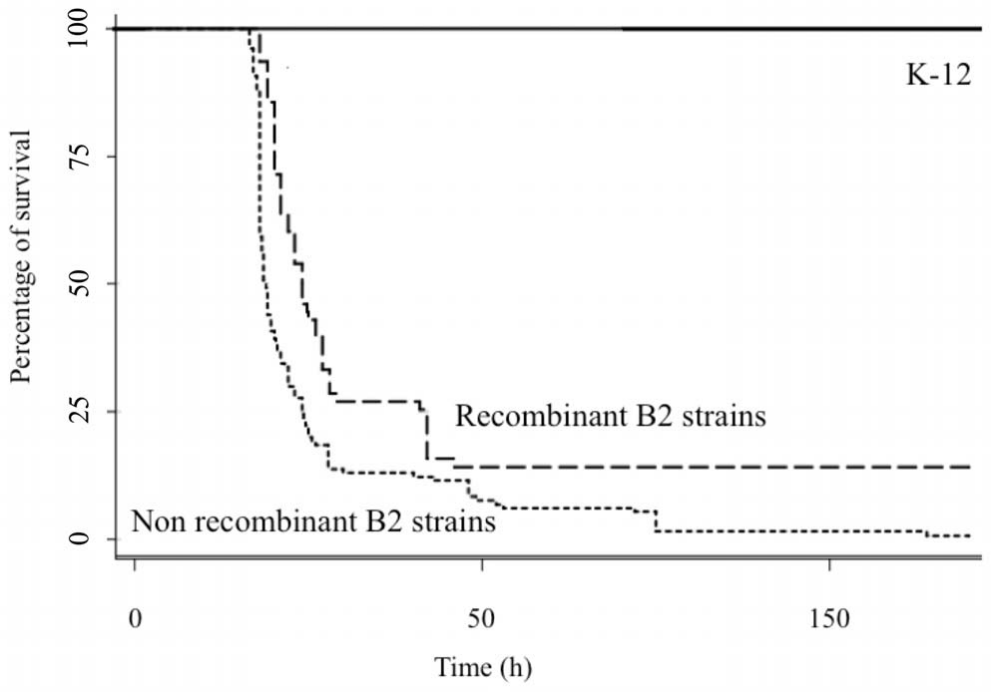
Recombinant *nhaA* B2 strains have a lower virulence. Lines represent the mean survival of OF1 mice after subcutaneous injection of 10^8^ cells of the following strains: solid line: K-12 MG1655; dotted line: B2 strains lacking recombination in *nhaA* (*i.e* CFT073, 536, S88, RS218) and dashed line: strains showing evidence of recombination in *nhaA* region (*i.e* ED1a, E2348/65, SE15, B671, H001, TA103, TA435).

Because some of the B2 strains with sign of recombination were known to be avirulent, we decided to study the intrinsic virulence of several B2 strains in the murine septicemia model. Among strains showing traces of recombination, we observed a significant decrease in lethality for ED1a [14], E2348/63 [14], SE15 (this work) but not for H001 (this work), TA435 (this work), and TA103 (this work) compared to other B2 strains responsible for extra intestinal infections. Indeed, we then compared the mean survival rate between strains with a recombinant *nhaAR* operon (ED1a, E2348/69, SE15, H001, TA103 and TA435) and strains with a non-recombinant *nhaAR operon* (CFT073, 536, F11, S88, APEC01, UTI89, LF82 and B2S) [14], [25]. We found a significant decrease in the intrinsic virulence of recombinant strains (p<0.0001) (Figure 5).

**Figure 6:**
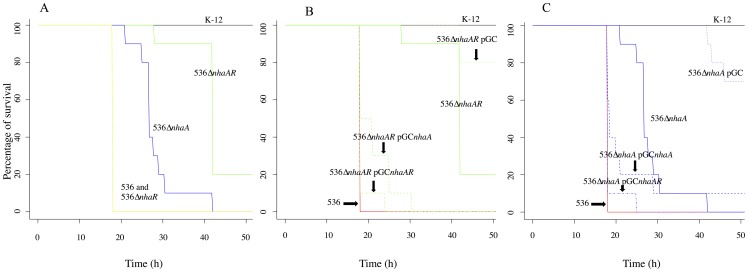
Impact of *nhaAR* operon on virulence. Lines represent the survival of OF1 mice after subcutaneous injection of 10^8^ cells of the following strains. In (A), (B) and (C) black solid lines, K-12 MG1655 and red solid lines, strain 536. In (A) orange, blue and green solid lines, mice injected with mutants 536Δ*nhaR*, 536Δ*nhaA* and 536Δ*nhaAR*, respectively. In B (C) dashed-dotted lines, complemented mutants 536Δ*nhaA* pGC*nhaAR* (536Δ*nhaAR* pGC*nhaAR*), dashed lines, complemented mutants 536Δ*nhaA* pGC*nhaA* (536Δ*nhaAR* pGC*nhaA*), solid lines, 536Δ*nhaA* (536Δ*nhaAR*) and dotted lines, complemented mutants 536Δ*nhaApGC* (536Δ*nhaAR* pGC).

We further tested the effect of the *nhaAR* deletion in this mice model, using 2 virulent B2 strains. 536Δ*nhaAR* showed a dramatically decreased lethality compared to wild-type 536 strain (p<0.001) (Figure 6). To confirm these results, we reproduced them in CFT073, another highly lethal B2 strain, and found similar results (data not shown). We also tested in this model the complemented strain 536Δ*nhaAR* pGC*nhaAR* and 536Δ*nhaAR* pGC. The comparison of 536Δ*nhaAR* pGC*nhaAR* with 536 strain (p = 0.48) and 536Δ*nhaAR* (p = 4.3e-06) proved that *nhaAR* operon was implicated in virulence and the comparison of 536Δ*nhaAR* pGC with 536Δ*nhaAR* (p<0.01) indicated a cost of the empty plasmid in this model, reinforcing the implication of *nhaAR* in the extra-intestinal virulence (Figure 6B). We also performed competition assays between wild type CFT073 and 536 strains and their isogenic mutant counterparts, CFT073Δ*nhaAR*:Cm and 536Δ*nhaAR*:Cm in the murine septicemia model. We used 5 mice in each group and the experiment was repeated one time (10 mice in each group, total). 536 and CFT073Δ*nhaAR* chloramphenicol-resistant cells were 5.33±1.14 and 3.06±1.4 orders of magnitude less numerous in the spleen than their wild type counterparts, respectively. In contrast, when Δ*nhaAR* and Δ*nhaAR*:Cm strains were injected together to the mice, no difference in spleen bacterial counts were noted, which is an indirect evidence for the absence of cost of the resistance marker *in vivo* (p = 0.51). To determine the implication of each gene of the operon *nhaAR*, we constructed and then tested the deleted mutant strains 536Δ*nhaA* and 536Δ*nhaR* in this model (Figure 6). We first determined using qRT-PCR of *nhaR* gene that in 536Δ*nhaR* strain, the gene *nhaR* was no longer expressed, and that, in 536Δ*nhaA*, *nhaR* was expressed (data not shown). In the mouse model 536Δ*nhaA* showed a significant attenuation of lethality compared to wild type 536 strains (p<0.001) whereas 536Δ*nhaR* did not show significant difference with 536 (Figure 6A). Complementation of deleted strains 536Δ*nhaA* and 536Δ*nhaAR* strains with pGC*nhaA* and pGC*nhaAR* allowed us to observe significant differentiations between 536Δ*nhaA* and 536Δ*nhaA* pGC*nhaAR* (p = 2.8E-5), 536Δ*nhaA* and 536Δ*nhaA* pGC*nhaA* in the 30 first hours (p<0.01), 536Δ*nhaAR* and 536Δ*nhaAR* pGC*nhaAR* as described above, 536Δ*nhaAR* and 536Δ*nhaAR* pGC*nhaA*. These observations lead us to conclude that complementation of *nhaA* deletion by achieved by either *nhaA* or *nhaAR* restored a high virulence (Figure 6B and C).

Hence, despite the fact that we could not find strong evidence of *nhaAR* phenotypic implication *in vitro*, it seems that the operon is critical in the mouse model of septicemia, and that the presence of the recombination in the operon is associated with a lower virulence. To go further in the *in vivo* characterization of *nhaAR* role, we finally tested strain 536 and 536Δ*nhaAR* in the murine model of gut colonization. No effect of the mutation was found in the colonization model after 7 days, both when strains were added separately (mean p value during 7 days 0.2) or together in competition (mean p value during 7 days 0.63). Here again controls showed no cost of the chloramphenicol marker (mean p value during 7 days 0.74). The effect of *nhaAR* deletion seems to be restricted to virulence conditions.

### Discussion

In modern biology, genomics is used to identify candidate genes associated with some phenotypes of interest. In microbiology, the genomic plasticity has lead scientists to use genomics-phenotype association mostly to focus on presence and absence of genes. In this paper, we tried to use the genomic approach to uncover some molecular determinants of B2 non-B2 differentiation using the sequence of the core genes. In that case, we lack a clear understanding of the phenotypes that may explain the difference of prevalence of these groups of strains as human commensals, and we thought that finding genes with marked difference between these groups might provide some hints. Using the fraction of fixed sites between the two groups, we identified *nhaA* as a clear outlier from the distribution. When we excluded some strains involved in recombination in the *nhaA* region, we found that this extreme pattern could be extended to the whole *nhaAR* operon.

How can such a pattern have emerged? When we looked at the *nhaAR* genomic environment we saw that the operon was flanked by highly volatile modules (Figure 1C). While the operon was conserved in all strains, flanking regions required multiple acquisition and loss to be compatible with the species phylogeny (Figure 2). Therefore, the large diversity between B2 and non-B2 could be due to the acquisition of an *nhaAR* operon by horizontal transfer. Several other observations support these hypotheses. The branch between B2 and non-B2 is not enriched in non-synonymous mutations as would be expected in the case of a strong selection. Moreover the diversity within B2 and non-B2 is not high (it is rather low indeed) which rejects a high local mutation rate.

Further investigation on the *nhaAR* sequences revealed some traces of recombination among the B2 strains (Figure 3). Interestingly some of the strains involved in these recombinations were atypical B2 in term of virulence. None of them were isolated in extra-intestinal virulence conditions and the ones tested in a mouse model of septicemia were remarkable by their lack of virulence. We therefore decided to investigate functionally the role of *nhaAR* operon diversity.

*nhaA* is coding for a sodium proton antiporter which is know to be responsible for pH and sodium homeostasis in *E. coli* [16], particularly it allows growth of bacterial cells in high pH and high sodium concentrations [33]. NhaA protein is a membranous protein allowing the exchange of 2 protons against a sodium ion [34]. Padan *et al.* also described that *nhaA* mutants were not able to growth at high pH, underlining the importance of this protein in these conditions [35]. Transcription of *nhaA* is dependent of two regulators, Hns acting as a repressor and NhaR that activates the expression of *nhaA*, but also other genes, *i.e.*, *pgaA* and *osmC* [36], [37]. *nhaR* is a central regulator of genes involved in stress responses (Figure 1A), *nhaA*, *pgaA* and *osmC* in high pH, high osmolarity, exposure to organic hydroperoxide or biofilm conditions [38]–[40]. *nhaR* is also regulated by the pleiotropic regulator CsrA in its upstream region. As high concentrations of Li + and Sodium ions [33] and high values of pH [32], [34] promote the activity of NhaA, we studied the expression patterns of *nhaAR* and *osmC* at different pH and osmolarities, but failed to detect significant differences between B2 and non-B2 strains using reporter plasmids.

As changes of expression may be too small to be detected or may occur at a specific timing, we then focused on integrated phenotypes such as growth curves in different media (complex and minimum medium) at different pH (neutral and high pH) and concentration of sodium (170 and 350 mmoL/L). We found a significant differentiation between B2 and non-B2 strains at high pH and high concentration of sodium (Figure 4). These conditions are the ones in which NhaA is supposed to be expressed. However, when we looked for the specific implication of *nhaAR* region using wild type and deleted mutant strains of B2 and non-B2 group, we did not observed any significant differentiations between B2 and non-B2 strains.

Because our laboratory conditions may not be the ones in which a differentiation is strongly expressed, we tried some *in vivo* assays. As a significant number of recombinant strains for *nhaAR* region (ED1a and E2348/69) are known to be avirulent in a murine model of septicemia, we tested this model for some other recombinants identified (SE15, TA103 and TA435). We found that among the recombinant strains half of the strains tested showed decreased or absence of virulence in the murine model of septicemia which is significantly different from the other B2 strains (Figure 5) which are known to be virulent in this model [14], [25]. This observation lead us to hypothesize that *nhaAR* could have implication in virulence or colonization process as it is now known that extra-intestinal and commensalism are linked [14]. We then tested two Δ*nhaAR* mutant strains belonging to the B2 phylogenetic group (CFT073 and 536) and observed an important decrease of the intrinsic virulence of the strains in this model completely complemented by a vector bearing *nhaAR* operon (Figure 6). Though, when we tested 536Δ*nhaAR* mutant in competition in a mouse gut colonization, we did not find any impact of the deletion. Hence, the effect of the mutation is only marked in the virulence model. Interestingly, the deletion of *nhaAR* operon seems to have a stronger impact on virulence than the deletion of the pathogenicity island (PAI) of strain 536 in isolations or in combination [13], [41]. While the single PAI deletions had no effect with similar inoculum as the one used here, the mutant with all 7 PAI deleted killed 50% of mice in 28 hours compared to 42 hours in Δ*nhaAR* and 18 h in 536.

How could *nhaAR* contributes to virulence? NhaR is a central regulator of expression of the genes *nhaA*, *osmC* and the operon *pgaABCD* involved in stress responses such as high salinity, high pH or biofilm formation [38]–[40]. Implication of these genes in the virulence process is not clear, except for *pgaABCD* which has been proved to be implicated in urinary tract ascending infections [42]. However we were not able to prove specific *nhaR* implication in this model. But we clearly showed implication of *nhaA* gene in this attenuation of virulence using deleted and complemented strains with this gene. NhaA is known to be responsible for growth of bacterial cells in high pH and high sodium concentrations [33], yet such conditions are not the ones that seem to prevail during sepsis where low pH seem to be dominant [43]. Further investigation will therefore be needed to fully understand the contribution of *nhaAR* to virulence.

### Conclusions

Through a bioinformatics approach we identified a candidate core gene involved in B2, non-B2 genetic differentiation. Many assays were performed to test some phenotypic expression of this diversity *in vitro* without a clear success. However, when we used *in vivo* experiments, though we only focused on the analysis of knock-outs, we found a strong and so far unnoticed implication of *nhaA* gene in virulence, despite a lack of effect in commensalism. This whole process illustrates that bioinformatics approaches may identify genes of interest whose effect is mostly if not only visible in complex *in vivo* environments.

## Supporting Information

Table S1 Strains and plasmids used in the *in vitro* and *in vivo* assays in this study.
(DOCX)

Table S2 List of primers used in this study.
(DOCX)

Table S3 List of conditions used in the growth curves experiments.
(DOCX)

Table S4 List of 128 genomes used in the study to identify markers of differentiation of the B2 phylogenetic group from other group.
(DOC)

Table S5 List of genes classified by the proportions of fixed differences between B2 and non-B2 between each gene of the core and the whole set of genes pooled together using libsequence [26].
(XLS)
