# Correction: The Conserved *nhaAR* Operon Is Drastically Divergent between B2 and Non-B2 *Escherichia coli* and Is Involved in Extra-Intestinal Virulence

**DOI:** 10.1371/journal.pone.0118729

**Published:** 2015-03-18

**Authors:** The PLOS ONE Staff

The ninth author’s name and affiliation are incorrect. The correct name is: Stephane Cruveiller. The correct affiliation is #4 Commissariat à l'énergie atomique et aux énergies alternatives (CEA), Institut de génomique, Génoscope, Laboratoire d'Analyses Bioinformatiques pour la Génomique et le Métabolisme (LABGeM), Evry, France.
