# Supplementary material for: The Conserved nhaAR Operon Is Drastically Divergent between B2 and Non-B2 Escherichia coli and Is Involved in Extra-Intestinal Virulence
Source: PLoS One. 2014 Sep 30;9(9):e108738. doi: 10.1371/journal.pone.0108738 (PMC4182557; doi:10.1371/journal.pone.0108738)
Supplement: Table S1 — Strains and plasmids used in the in vitro and in vivo assays in this study. (DOCX) [file pone.0108738.s001.docx]

| Strain or plasmid | Phylogenetic group | Description or genotype | Source or reference |
| --- | --- | --- | --- |
| *E. coli* K-12 strains | A |  |  |
| K-12 MG1655 | A |  | [44] |
| K-12Δ*nhaAR*:Cm | A | *cat* | This study |
| K-12Δ*nhaAR* | A |  | This study |
| K-12 p*nhaA* | A | *kan* | This study |
| K-12Δ*nhaAR:Cm* p*nhaA* | A | *cat, kan* | This study |
| K-12 p*osmC* | A | *kan* | This study |
| K-12Δ*nhaAR*:Cm p*osmC* | A | *cat, kan* | This study |
| IAI1 strains | B1 |  |  |
| IAI1 wild type strain | B1 |  | [1] |
| IAI1Δ*nhaAR*:Cm | B1 | *cat* | This study |
| IAI1Δ*nhaAR* | B1 |  | This study |
| IAI1 p*nhaA* | B1 | *kan* | This study |
| IAI1Δ*nhaAR:Cm* p*nhaA* | B1 | *cat, kan* | This study |
| IAI1 p*osmC* | B1 | *kan* | This study |
| IAI1Δ*nhaAR*:Cm p*osmC* | B1 | *cat, kan* | This study |
| TA249 strains |  |  |  |
| TA249 wild type strain | D |  | Gordon D; http://www.broadinstitute.org |
| TA249Δ*nhaAR*:Cm | D | *cat* | This study |
| TA249Δ*nhaAR* | D |  | This study |
| TA249 p*nhaA* | D | *kan* | This study |
| TA249Δ*nhaAR:Cm* p*nhaA* | D | *cat, kan* | This study |
| TA249 p*osmC* | D | *kan* | This study |
| TA249Δ*nhaAR*:Cm p*osmC* | D | *cat, kan* | This study |
| 536 strains | B2 |  |  |
| 536 wild type strain | B2 |  | [45] |
| 536Δ*nhaAR*:Cm | B2 | *cat* | This study |
| 536Δ*nhaAR* | B2 |  | This study |
| 536 Δ*nhaR* | B2 |  | This study |
| 536 Δ*nhaA* | B2 |  | This study |
| 536Δ*nhaAR:Cm* p*nhaA* | B2 | *cat, kan* | This study |
| 536 p*osmC* | B2 | *kan* | This study |
| 536Δ*nhaAR:Cm* p*osmC* | B2 | *cat, kan* | This study |
| 536Δ*nhaAR* pGC*nhaAR* | B2 | *kan* | This study |
| 536Δ*nhaAR* pGC*nhaA* | B2 | *kan* | This study |
| 536Δ*nhaAR* pGC | B2 | *kan* | This study |
| 536Δ*nhaA* pGC*nhaAR* | B2 | *kan* | This study |
| 536Δ*nhaA* pGC*nhaA* | B2 | *kan* | This study |
| 536Δ*nhaA* pGC | B2 | *kan* | This study |
| CFT073 strains | B2 |  |  |
| CFT073 wild type strain | B2 |  | [46] |
| CFT073Δ*nhaAR*:Cm | B2 | *cat* | This study |
| CFT073Δ*nhaAR* | B2 |  | This study |
| CFT073 p*nhaA* | B2 | *kan* | This study |
| CFT073Δ*nhaAR:Cm* p*nhaA* | B2 | *cat, kan* | This study |
| CFT073 p*osmC* | B2 | *kan* | This study |
| CFT073Δ*nhaAR:Cm* p*osmC* | B2 | *cat, kan* | This study |
| TA014 strains | B2 |  |  |
| TA014 wild type strain | B2 |  | Gordon D; http://www.broadinstitute.org |
| TA014Δ*nhaAR*:Cm | B2 | *cat* | This study |
| TA014Δ*nhaAR* | B2 |  | This study |
| TA014 p*nhaA* | B2 | *kan* | This study |
| TA014Δ*nhaAR:Cm* p*nhaA* | B2 | *cat, kan* | This study |
| TA014 p*osmC* | B2 | *kan* | This study |
| TA249Δ*nhaAR*:Cm p*osmC* | B2 | *cat, kan* | This study |
| EC7372 | B2 |  | [47] |
| ECOR54 | B2 |  | [47] |
| IAI73 | B2 |  | [47] |
| F11 | B2 |  | [47] |
| Asp12e | B2 |  | [47] |
| S107 | B2 |  | [47] |
| J96 | B2 |  | [47] |
| C1845 | B2 |  | [47] |
| RS218 | B2 |  | [51] |
| ED1a | B2 |  | [47] |
| SE15 | B2 |  | [26] |
| E2348/69 | B2 |  | [47] |
| HS | A |  | [47] |
| TA007 | A |  | Gordon D; http://www.broadinstitute.org |
| 55989 | B1 |  | [47] |
| AD6 | B1 |  | [10] |
| 042 | D | *cat* | [47] |
| ECOR46 | D |  | [47] |
| EDL933 | E |  | [47] |
| IAI39 | D |  | [47] |
| ECOR41 | D |  | [47] |
| ECOR70 | C |  | [47] |
| Plasmids |  |  |  |
| pKD3 |  | *cat* | [17] |
| pKD46 |  | For arabinose induction of λ Red system | [17] |
| pCP20 |  | For FLP-recombinase production | [17] |
| p*nhaA* |  | Promoter region of *nhaA* placed upstream *gfp, kan* | [22] |
| p*osmC* |  | Promoter region of *osmC* placed upstream *gfp, kan* | [22] |
| pGC |  | pSMART GC LK vector used for complementation, *kan* | [48] |
