# Supplementary material for: The Conserved nhaAR Operon Is Drastically Divergent between B2 and Non-B2 Escherichia coli and Is Involved in Extra-Intestinal Virulence
Source: PLoS One. 2014 Sep 30;9(9):e108738. doi: 10.1371/journal.pone.0108738 (PMC4182557; doi:10.1371/journal.pone.0108738)
Supplement: Table S2 — List of primers used in this study. (DOCX) [file pone.0108738.s002.docx]

| Name of primer | Sequence (5’-3’) | Targeted sequence of amplification or deletion | Source or reference |
| --- | --- | --- | --- |
| WanF_nonB2_nhaAR | ACTTTTCCTAATTAATCCCTCAGGAATCCTCACCTTAAGCTATGATTATCTGTGTAGGCTGGAGCTGCTTC | *nhaAR* operon in non-B2 strains | This study |
| WanR_K12TA249_nhaAR | AATGTACAACTAATCTATCTCCCCCACCTTAACTTTGGGGGAGCTGCCGACATATGAATATCCTCCTTAG | *nhaAR* operon in K-12 and TA249 strains | This study |
| WanR_IAI1_nhaAR | TATGTACAACTAATCTATCTCCCCCACCTTAACTTTGGGGGGAGGTGCCGACATATGAATATCCTCCTTAG | *nhaAR* operon in IAI1 strains | This study |
| WanF_B2_nhaAR | GAATAATTTCTTCAGAAATCTTGACATTAGACTATGATTAATGGGGTTTATGTGTAGGCTGGAGCTGCTTC | *nhaAR* operon in B2 strains | This study |
| WanR_B2_nhaAR | TTTTTTTATATCCATCTGCAGGCCAAAATTCGGCCTGCAAAGTACAAATGTCATATGAATATCCTCCTTAG |  | This study |
| WanF_B2_nhaA | TGCGGGGTAAAATCGTGAAAACGATCTATTCACCTGAAGAGAAATAAAAAGTGTAGGCTGGAGCTGCTTC | *nhaA* in B2 strains | This study |
| WanR_B2_nhaA | GATAACAATGAAAAGGGAGCCGTTTATGGCTCCCCAGTACATCGTCCTGCATATGAATATCCTCCTTAG |  | This study |
| WanF_B2_nhaR | GTACTGGGGAGCCATAAACGGCTCCCTTTTCATTGTTATCAGGGAGAGAATGTGTAGGCTGGAGCTGCTTC | *nhaR* in B2 strains | This study |
| verifWanF_nonB2_nhaAR | CAAGCATCGCCGACTGACAA | *nhaAR* operon in non-B2 strains | This study |
| verifR_nonB2_nhaAR | GCAATTACTACTCTTTTCAGC |  | This study |
| verifWanF_B2_nhaAR | GGAATAAAAAACTAGAGCATGG | *nhaAR* operon in B2 strains | This study |
| verifR_B2_nhaAR | TCAGGCTGCTAAAGGTCTGA |  | This study |
| c1 | TTATACGCAAGGCGACAAGG | *cat* | [17] |
| c2 | GATCTTCCGTCACAGGTAGG |  | [17] |
| cp_536F | AATGTCTCCTGCGAATGTCG | *nhaAR* operon containing promoter region in 536 strain | This study |
| cp_536R | TGCCTGTTGTTGTCAGCTTTG |  | This study |
| cp_536DnhaA_R | CCGGTAATGGTTTGTGGTGT | *nhaA* containing promoter region in 536 strain | This study |
| qRT_nhaR__F | TCTGGTCAGCAGTGTTCTGG | *nhaR* | This study |
| qRT_nhaR_R | CCTTCCTGCTGAGTGGAATC |  | This study |
| ECFW | CATGCCGCGTGTATGAAGAA | 16S *rRNA* gene | [49] |
| ECRV | CGGGTAACGTCAATGAGCAAA |  | [49] |
