# Supplementary material for: The Conserved nhaAR Operon Is Drastically Divergent between B2 and Non-B2 Escherichia coli and Is Involved in Extra-Intestinal Virulence
Source: PLoS One. 2014 Sep 30;9(9):e108738. doi: 10.1371/journal.pone.0108738 (PMC4182557; doi:10.1371/journal.pone.0108738)
Supplement: Table S3 — List of conditions used in the growth curves experiments. (DOCX) [file pone.0108738.s003.docx]

| LB | pH 7 | | pH 8.5 | |
| --- | --- | --- | --- | --- |
|  | Na 170 mmol/L | Na 350 mmol/L | Na 170 mmol/L | Na 350 mmol/L |
| Minimum medium Davis | pH 7 | | pH 8 | |
|  | Na 170 mmol/L | Na 350 mmol/L | Na 170 mmol/L | Na 350 mmol/L |
