# Supplementary material for: The Conserved nhaAR Operon Is Drastically Divergent between B2 and Non-B2 Escherichia coli and Is Involved in Extra-Intestinal Virulence
Source: PLoS One. 2014 Sep 30;9(9):e108738. doi: 10.1371/journal.pone.0108738 (PMC4182557; doi:10.1371/journal.pone.0108738)
Supplement: Table S4 — List of 128 genomes used in the study to identify markers of differentiation of the B2 phylogenetic group from other group. (DOC) [file pone.0108738.s004.doc]

| Strain name | *Escherichia coli* group or *Escherichia* clade | GenBank ID, GenBank Assembly ID or website reference |
| --- | --- | --- |
| 536 | B2 | CP000247 |
| APEC O1 | B2 | CP000468 |
| B108 | B2 | http://www.broadinstitute.org |
| B671 | B2 | http://www.broadinstitute.org |
| CFT073 | B2 | 26111730 |
| E2348/69 | B2 | FM180568 |
| ED1a | B2 | CU928162 |
| F11 | B2 | GCA_000167835 |
| H001 | B2 | http://www.broadinstitute.org |
| H223 | B2 | http://www.broadinstitute.org |
| H252 | B2 | http://www.broadinstitute.org |
| H263 | B2 | http://www.broadinstitute.org |
| H296 | B2 | http://www.broadinstitute.org |
| H305 | B2 | http://www.broadinstitute.org |
| H378 | B2 | http://www.broadinstitute.org |
| H413 | B2 | http://www.broadinstitute.org |
| H461 | B2 | http://www.broadinstitute.org |
| H504 | B2 | http://www.broadinstitute.org |
| H588 | B2 | http://www.broadinstitute.org |
| H660 | B2 | http://www.broadinstitute.org |
| LF82 | B2 | CU651637 |
| M605 | B2 | GCA_000176555 |
| R527 | B2 | http://www.broadinstitute.org |
| S88 | B2 | CU928161 |
| SE15 | B2 | AP009378 |
| TA014 | B2 | http://www.broadinstitute.org |
| TA103 | B2 | http://www.broadinstitute.org |
| TA206 | B2 | GCA_000176595 |
| TA435 | B2 | http://www.broadinstitute.org |
| TA464 | B2 | http://www.broadinstitute.org |
| UTI89 | B2 | CP000243 |
| 42 | non-B2 | FN554766 |
| 53638 | non-B2 | CP001064 |
| 55989 | non-B2 | CU928145 |
| 101-1 | non-B2 | GCA_000168095 |
| ATCC 8739 | non-B2 | CP000946 |
| B088 | non-B2 | GCA_000163155 |
| B171 | non-B2 | http://www.broadinstitute.org |
| B175 | non-B2 | http://www.broadinstitute.org |
| B185 | non-B2 | http://www.broadinstitute.org |
| B354 | non-B2 | http://www.broadinstitute.org |
| B367 | non-B2 | http://www.broadinstitute.org |
| B574 | non-B2 | http://www.broadinstitute.org |
| B706 | non-B2 | http://www.broadinstitute.org |
| B921 | non-B2 | http://www.broadinstitute.org |
| BL21 GOLD | non-B2 | CP001665 |
| BREL 606 | non-B2 | CP000819 |
| DH1 | non-B2 | CP001637 |
| E1002 | non-B2 | http://www.broadinstitute.org |
| E110019 | non-B2 | GCA_000167875 |
| E1114 | non-B2 | http://www.broadinstitute.org |
| E1167 | non-B2 | GCA_000190795 |
| E1520 | non-B2 | http://www.broadinstitute.org |
| E22 | non-B2 | GCA_00016785 |
| E24377A | non-B2 | CP000800 |
| E267 | non-B2 | http://www.broadinstitute.org |
| E482 | non-B2 | http://www.broadinstitute.org |
| E560 | non-B2 | http://www.broadinstitute.org |
| E704 | non-B2 | http://www.broadinstitute.org |
| ETEC H10407 | non-B2 | FN649414 |
| FVEC 1302 | non-B2 | GCA_000163215 |
| FVEC 1412 | non-B2 | GCA_000163235 |
| FVEC 1465 | non-B2 | http://www.broadinstitute.org |
| H120 | non-B2 | http://www.broadinstitute.org |
| H185 | non-B2 | http://www.broadinstitute.org |
| H218 | non-B2 | http://www.broadinstitute.org |
| H220 | non-B2 | http://www.broadinstitute.org |
| H288 | non-B2 | http://www.broadinstitute.org |
| H299 | non-B2 | http://www.broadinstitute.org |
| H383 | non-B2 | http://www.broadinstitute.org |
| H386 | non-B2 | http://www.broadinstitute.org |
| H420 | non-B2 | http://www.broadinstitute.org |
| H454 | non-B2 | http://www.broadinstitute.org |
| H489 | non-B2 | http://www.broadinstitute.org |
| H591 | non-B2 | http://www.broadinstitute.org |
| H593 | non-B2 | http://www.broadinstitute.org |
| H617 | non-B2 | http://www.broadinstitute.org |
| H736 | non-B2 | http://www.broadinstitute.org |
| HS | non-B2 | CP000802 |
| IAI1 | non-B2 | CU928160 |
| IAI39 | non-B2 | CU928164 |
| K-12 | non-B2 | U00096 |
| K-12 DH10B | non-B2 | CP000948 |
| M056 | non-B2 | http://www.broadinstitute.org |
| M114 | non-B2 | http://www.broadinstitute.org |
| M646 | non-B2 | http://www.broadinstitute.org |
| M718 | non-B2 | http://www.broadinstitute.org |
| O103:H2 12009 | non-B2 | AP010958 |
| O104:H4 LB226692 | non-B2 | CP003289 |
| O111:H1 11128 | non-B2 | AP010960 |
| O157:H7 EC4042 | non-B2 | GCA_000181775 |
| O157:H7 EC4045 | non-B2 | GCA_000181755 |
| O157:H7 EC4115 | non-B2 | CP001164 |
| O157:H7 EC4206 | non-B2 | GCA_000181775 |
| O157:H7 EDL933 | non-B2 | AE005174 |
| O157:H7 sakaï | non-B2 | BA00000 |
| O157:H7 TW14588 | non-B2 | CP001368 |
| O26:H1 111368 | non-B2 | AP010953 |
| PUTI 459 | non-B2 | http://www.broadinstitute.org |
| R424 | non-B2 | http://www.broadinstitute.org |
| R529 | non-B2 | http://www.broadinstitute.org |
| SE11 | non-B2 | AP009240 |
| T408 | non-B2 | http://www.broadinstitute.org |
| T426 | non-B2 | http://www.broadinstitute.org |
| TA007 | non-B2 | GCA_000190975 |
| TA008 | non-B2 | http://www.broadinstitute.org |
| TA024 | non-B2 | http://www.broadinstitute.org |
| TA054 | non-B2 | http://www.broadinstitute.org |
| TA141 | non-B2 | http://www.broadinstitute.org |
| TA143 | non-B2 | GCA_000176615 |
| TA144 | non-B2 | http://www.broadinstitute.org |
| TA249 | non-B2 | http://www.broadinstitute.org |
| TA255 | non-B2 | http://www.broadinstitute.org |
| TA271 | non-B2 | GCA_000176635 |
| TA280 | non-B2 | GCA_000176655 |
| TA447 | non-B2 | http://www.broadinstitute.org |
| UMN026 | non-B2 | CU928163 |
| W3110 | non-B2 | AP009048 |
| B7a | non-B2 | GCA_000167815 |
| DAV92 | non-B2 | http://www.genoscope.cns.fr/agc/microscope/home/index.php |
| O104:H4 ty-2482 | non-B2 | GCA_000217695 |
| E1118 | *Escherichia* clade V | http://www.broadinstitute.org |
| E1492 | *Escherichia* clade I | http://www.broadinstitute.org |
| H442 | *Escherichia* clade I | http://www.broadinstitute.org |
| H605 | *Escherichia* clade IV | http://www.broadinstitute.org |
| M863 | *Escherichia* clade I | http://www.broadinstitute.org |
| TA004 | *Escherichia* clade III | http://www.broadinstitute.org |
| TW10509 | *Escherichia* clade I | http://www.broadinstitute.org |
